# The effect of seasonal thermal stress on milk production and milk compositions of Korean Holstein and Jersey cows

**DOI:** 10.5713/ajas.19.0926

**Published:** 2020-05-12

**Authors:** Dong-Hyun Lim, Vijayakumar Mayakrishnan, Kwang-Seok Ki, Younghoon Kim, Tae-Il Kim

**Affiliations:** 1 Dairy Science Division, National Institute of Animal Science, Rural Development Administration, Cheonan 31000, Korea; 2 Department of Agricultural Biotechnology and Research Institute of Agriculture and Life Science, Seoul National University, Seoul 08826, Korea

**Keywords:** Milk Production, Season, Temperature-humidity Index, Milk Composition

## Abstract

**Objective:**

In this study we investigated the effect of seasonal thermal stress on milk production and milk compositions between Holstein and Jersey dairy cows under the temperate-climate in Korea.

**Methods:**

A total of 9 Holstein lactating dairy cows (2.0±0.11 parity) which had a daily milk yield of 29.77±0.45 kg, and days in milk of 111.2±10.29 were selected similarly at the beginning of the experiments in each season. Also, a total of 9 Jersey lactating dairy cows (1.7±0.12 parity) which had a daily milk yield of 20.01±0.43 kg, and days in milk of 114.0± 9.74 were selected similarly at the beginning of the experiments.

**Results:**

Results showed that the average ambient temperature (°C) and temperature-humidity index (THI) were higher in summer, and were lower in winter (p<0.05). The average relative humidity (RH, %) was higher in autumn than that of other seasons (p<0.05). Milk production was significantly decreased (Holstein 29.02 kg/d and Jersey 19.75 kg/d) in autumn than in other seasons (Holstein 30.14 kg/d and Jersey 20.96 kg/d). However, the milk production was negatively correlated in Holstein cows, and positively correlated in Jersey cows with THI values increased from 16 to 80. In addition, milk yield was increased by 15% in Holstein cows and decreased by 11% in Jersey cows with the THI values increased from 16 to 20. The fat and protein content percentage was significantly higher in Jersey milk than in Holstein milk, furthermore the fat and protein content yield was higher in Jersey cow milk than that of Holstein cow’s milk with all THIs.

**Conclusion:**

From the study results, we concluded that Jersey cows might be less adaptable to low temperature of the winter, and this would have a negative impact on dairy farmer income since Korea’s milk price estimation system places a higher value on milk yield than on milk compositions or sanitary grades.

## INTRODUCTION

Climate change is transforming the earth ecosystems, and such change is producing a series of damaging abnormal weather phenomena, with the summer prolonged and the temperature relatively increasing in the world [1]. Early research demonstrated that the increasing trend of mean/max/min surface temperature is evident during the summer season in Korea [2].

Heat stress (HS) is one of the most significant challenges to milk production facing dairy farmers during the summer. Climatic conditions in the Korean peninsula are such that the summer season is relatively hot, and generally accompanied by high relative humidity (RH). Thus HS is chronic in nature, there is often little relief from the heat during the evening hours, and intense bursts of combined temperature and humidity further depress performance. Lactating dairy cows produce a large quantity of metabolic heat and accumulate additional heat from radiant energy. Production and accumulation of heat, coupled with compromised cooling capability because of environmental conditions, causes heat load in the cow to increase to the point that body temperature rises, intake declines and ultimately the cow’s milk productivity declines [3].

In addition, as breeders select for higher milk production, they can mistakably also select for cows with less heat tolerance. Jersey breed is the second most important dairy breed in the world, the Jersey breed is smaller than Holstein breed, and their feed consumption is less than that of Holstein cows [4]. Besides, the faecal output, carbon gases emission, and space need for the breeding of Jersey cows are lower than that of Holstein cows [4,5]. Therefore, Jersey breeding is relatively beneficial to dairy farmers. Early research also demonstrated that as measured by milk yield Jersey cows might have more heat tolerance than Holstein cows [6]. For all these reasons, the dairy farmers and industries have a growing interest in raising Jersey breed. However, the relationship between breed and responses to seasonal HS has not been studied in Korea. Therefore, the objective of the current experiment was to evaluate the effects of seasonal changes on milk production and milk composition of Jersey breed compared with Holstein breed in Korea.

## MATERIALS AND METHODS

### Animals, management and experimental design

The study was conducted at the Department of Animal Resources Development, National Institute of Animal Science (NIAS), Cheonan, Republic of Korea (36°55′53.2″ North, 127°06′22.1″ East, 23 m altitude). All dairy cows were maintained as stated in standard guidelines, and the experimental protocol involved in this research was approved by the Institutional Animal Care and Use Committee (IACUC) at National Institute of Animal Science, Jeonju, Republic of Korea. A total of 9 Holstein lactating dairy cows (2.0±0.11 parity) which had a daily milk yield of 29.77±0.45 kg, and days in milk of 111.2±10.29 were selected similarly at the beginning of each season. Also, a total of 9 Jersey lactating dairy cows (1.7±0.12 parity) which had a daily milk yield of 20.01±0.43 kg, and days in milk of 114.0±9.74 were selected similarly at the beginning of the season. The experiment was carried out from January 2016 to July 2017. The cows were raised under the same management and environmental conditions and housed in an open loose barn. The loose open barn was designed with the overshot roof of a ridge exhaust, fans to move and exchange the air in summer, and winch-curtain to block the cold wind in winter. The total mixed ration (TMR) was offered once a day at 09:00 h. The composition of the TMR remained the same throughout the year and included corn silage 28.1%, mixed hay 14.6%, alfalfa hay 8.3%, concentrate mixture 39.1%, soybean meal 4.4%, corn grain 4.4%, and mineral and energetic components 1.0%. The TMR contained, on average, 47.3% dry matter (DM), 16.6% crude protein (CP), 4.96% ether extract (EE), 38.7% neutral detergent fiber (NDF), and 19.5% acid detergent fiber (ADF) on a DM basis. The percentages of DM, CP, and crude fiber in oat silage were 28.5%, 6.7%, and 35.1% and 30, 7.11%, and 34% for the spring and summer periods, respectively. The energy content of feed rations of all cows was 1.7 Mcal net energy of lactation/kg DM and total digestible nutrients 68.7%. TMR samples were taken monthly and stored at −20°C until analysis. Feeding was allowed throughout the 24-hour period. Drinking water was made available at all times.

### Measurement, sampling, and laboratory analysis

The ambient temperature (Ta, °C) and RH (%) was monitored with a thermo-hygrometer (Testo 174H, Testo Inc., West Chester, PA, USA) with an accuracy of ±0.5°C, and ±3% RH. The thermo-hygrometer was set to record every day per 30 minutes and placed in the feeding area at a height of 2 meters above the ground. The temperature and humidity values were used to calculate several THI values; THI was calculated for each 30 min temperature and humidity measurement according to the formula: THI = (0.8×°C)+(RH %×[°C−14.4])+46.4 [7]. The mean daily THI forms the mean of all available 30 minutes THI’s per day. The maximum THI values for everyday were calculated according to the same formula of Fox and Tylutki [8], with the maximal temperature and the minimum RH/d, and the minimum THI values for everyday were calculated with the minimum temperature and the maximum RH/d.

TMR samples were analyzed for DM, CP, and EE by AOAC (1995) procedure, and NDF and ADF by Van Soest et al [9]. The cows were milked two times a day (06:00 and 17:00 h) and milk yield of the individual cows was recorded at each milking on all experimental periods. Milk samples were collected from each cow twice daily at morning (06:00 h) and afternoon (17:00 h) biweekly, and was analyzed for protein, fat, and somatic cells using with a LactoScop (MK2, Delta Instruments, Drachten, the Netherlands).

Milk yield and milk protein and fat percentage were used to calculate the fat protein corrected milk (FPCM) yield [10]. The yields of milk fat and protein were calculated from milk yield and the contents of milk fat and protein, respectively.

### Statistical analysis

The data analysis was performed with the statistical package SAS Enterprise Guide 7.1 (SAS Institute Inc., Cary, NC, USA). Seasons were defined as follows: spring (March to May), summer (June to August), autumn (September to November), and winter (December to February). The THI measured during the experimental period was divided into 13 classes based on the daily mean as follows: 16 to 20, 21 to 25, 26 to 30, 31 to 35, 36 to 40, 41 to 45, 46 to 50, 51 to 55, 56 to 60, 61 to 65, 66 to 70, 71 to 75, and 76 to 80.

The effects of the THIs and seasons on the milk production and milk compositions of each breed (Holstein and Jersey cows) were analyzed by using the one-way analysis of variance (ANOVA). Tuckey’s test was used to separate the means when significance was indicated. The effects of a breed and THI, or breed and season on the milk production and milk composition were analyzed by using the mixed procedure with a restricted maximum likelihood model. The breed, THI or season, and their interactions were included as fixed effects. For comparisons between breeds, THIs or seasons, and breed×THIs or seasons, the p-values after Bonferroni post hoc test adjustment are presented. The values are presented as least-squares means and standard errors of the means unless otherwise stated. Differences were considered significant if a probability (p) of <0.05 was found, and trends are discussed for variables with p≤0.10.

## RESULTS

Meteorological data including the mean minimum and maximum Ta, RH, and THI measured during milk recording of each breed dairy cows in period from 2016 to 2017 are summarized in Table 1. The recorded average daily Ta and THI were higher in summer (24.02°C±0.05°C, 72.55±0.09), and was lower in winter (−1.62°C±0.09°C, 35.11±0.12) than that of all other seasons (p<0.05). The results showed that the average RH was significantly highest in autumn (72.51%) and was significantly lowest in spring season (59.75%) (p<0.05). The average daily THI ≥72 was observed to be 0.96% in the spring, 57.78% in summer, and 2.11% in the autumn, which indicates that cows were exposed to the critical point of HS. In contrast, cows experienced the cold stress (THI ≤38) by 0.48% in the spring, 2.82% in the autumn, and 68.86% in the winter season.

**Figure 1:**
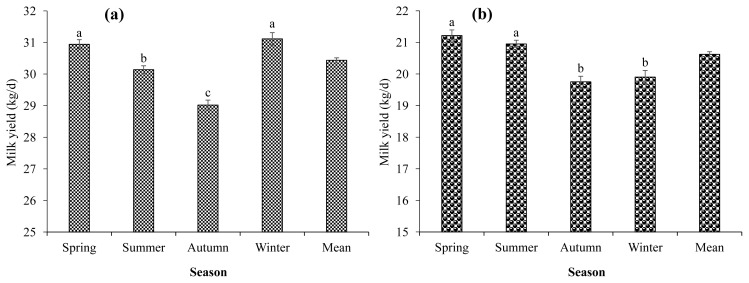
Milk yield of Holstein and Jersey cows against seasonal thermal changes. In Holstein cows, the lowered milk production (a) was observed during autumn season as compared to other seasons. In Jersey cows, the lowered milk production (b) was noted during autumn and winter when compared to other seasons. Values are expressed as mean±standard error of the mean. ^a,b^ Means with different superscripts in the row indicate significant differences (p<0.05).

The seasonal thermal stress effects on milk yield and FPCM yield trend between the Holstein and Jersey cows are represented in Table 2 and Figure 1. In Holstein dairy cows, milk production was decreased from the spring (30.94 kg/d) to autumn (29.02 kg/d), and then increased to winter season (31.12 kg/d) (p<0.05). However, in Jersey cows, milk production was lower in autumn (19.75 kg/d) and winter (19.90 kg/d) season than that of spring (21.22 kg/d) and summer season (20.96 kg/d) (p<0.05).

**Figure 2:**
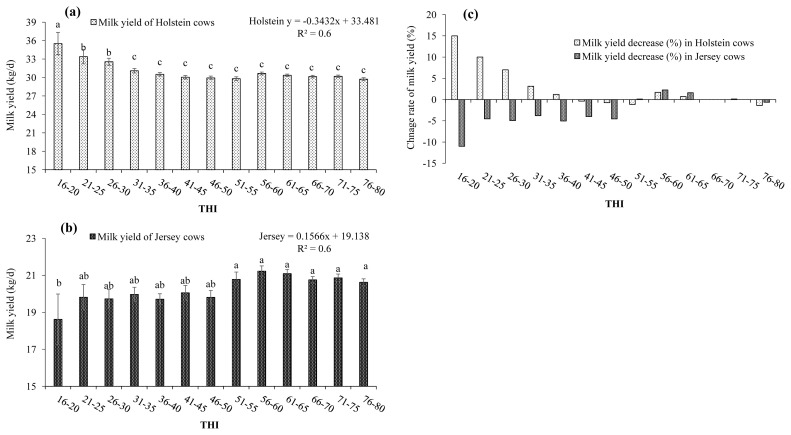
Milk yield and change rate of Holstein and Jersey cows against temperature-humidity index (THI) in Korean seasonal thermal changes. The milk production was negatively correlated in Holstein cows (a), and positively correlated in Jersey cows (b) with THI values increased from 16 to 80. The milk yield was increased by 15% in Holstein cows and decreased by 11% in Jersey cows as the THI values increased from 16 to 80 compared with the THI of 66 to 70 (c). Values are expressed as mean±standard error of the mean. ^a–c^ Means with different superscripts in the row indicate significant differences (p<0.05).

Results in Figure 2 show that the milk production of Holstein was highest (35.52 kg/d) at the 16 to 20 among the all THI ranges, then started to decline and remained constant (29.74 to 31.10 kg/d) when the THI values increased from 31 to 80 (p<0.05). On the contrary, the milk yield of Jersey cows was lowest (18.62 kg/d) at the 16 to 20 among the all THI ranges, and then the milk yield increased (20.63 to 21.22 kg/d) with a THI of 51 to 80 (p<0.05).

The present study has shown the relationship between the milk yield and THI values in Holstein and Jersey cows (Table 3). The average milk yield in Holstein and Jersey cows was decreased by 1% with a THI of 76 to 80. The average milk yield in Holstein cows was increased by 7% to 15% when the THI values decreased from 26–30 to 16–20. However, milk yield for Jersey cows dropped by 4% to 5% for THI values between 21 and 50, and reduced by 11% when the range of THI is 16 to 20. But the average milk yield was not affected by HS between the Holstein and Jersey cows with a THI value of 66 to 70.

The seasonal thermal stress effects on milk composition between the Holstein and Jersey cows are represented in Table 4. The study results showed that the milk fat percentage was higher in the winter (Holstein 4.19%, Jersey 5.62%) among the seasons in all breed’s milk (p<0.05). In Jersey cows, the milk fat percentage was lower (4.93%, p<0.05) in summer than in the other season. However, the milk protein percentage of Holstein cows gradually increased from the spring (3.21%) to the winter (3.44%) (p<0.05). However, the milk protein percentage of Jersey cows was increased more in autumn (3.88%) and winter (4.00%) than in spring (3.75%) and in summer (3.52%) (p<0.05). In Holstein cows, somatic cell count (SCC) was significantly increased in the summer (160.46×10^3^/mL). But, in Jersey cow, SCC was significantly higher in the autumn (275.07×10^3^/mL).

The effects of THI on the yield of milk fat and protein of Holstein and Jersey cows are presented in Table 5. The milk fat and protein yields were highest in Holstein cows (1.30 to 1.32 kg/d and 1.11 to 1.12 kg/d, respectively) when the THI values increased from 21 to 30, and were lowest (1.09 kg/d and 0.93 kg/d, respectively) when the THI ranged from 46 to 50 among the all THI ranges of 21 to 80 (p<0.05). In Jersey cows, yield of milk fat was highest (1.15 kg/d) at the THI of 31 to 35, milk protein was highest (0.79 kg/d) at THI of 26 to 30 of THI, and lowest milk fat and protein yield was recorded (1.00 kg/d and 0.71 kg/d) at a THI of 76 to 80 (p<0.05).

## DISCUSSION

The average daily Ta (°C) and THI were significantly higher in summer and lower in winter when measured data from each seasonal period were analyzed (p<0.05). However, the average RH (%) was highest in autumn (p<0.05). The THI represents the combination of Ta and RH, and used as a comprehensive indicator to assess the heat and cold stress degree of dairy cows. Some studies have explored the thermoneutral zone which is between 5°C and 25°C [11], and is within the range of −0.5°C to 20.0°C Ta and 60% to 80% RH [12]. When THI exceeds 72, the cows begin to experience HS, while when THI is less than 38, the cows begin to experience cold stress [13]. Among our study results, the average daily Ta exceeded 20°C on 8.65% of the time in the spring, 92.00% in the summer, and 24.65% in the autumn season. Also, average daily Ta ≤5 was recorded on 10.10% in the spring, 11.97% in the autumn, and 96.41% in the winter season. The average RH of >80% were higher in summer (23.21%) and autumn (23.94%), compared with the spring (7.21%) and winter (13.17%) season. The study results confirmed that the cows may suffer from both the heat and cold stress in spring and autumn season, particularly cows experienced the stress with higher RH (>80%) in autumn season than in spring season.

The average milk yield was reduced by 6.2% and 6.9% in Holstein and Jersey cows respectively as the average THI values changed from the spring to the autumn season and this lowered level may be due to the energy expenditure. Our study results are supported by Johnson et al [14] who reported early that the greater heat production can explain the increasing rate of decline in milk yield for cows. Also, the same authors explained that the permanent drop in the current lactation is proportional to the length of the HS. This study is consistent with earlier findings of Bohmanova et al [15] that the decline in milk production due to HS occurs with higher temperature in a semi-arid climate (≥30°C, 25% RH) than in a humid climate (≥23°C, 75% RH).

Berman [16] reported that the higher milk yield is associated with HS, which by itself would shift lower critical temperature (LCT) to lower ambient temperatures. The LCT decreases in −4°C [17] to values of −30°C during peak lactation [18], −37°C to −16°C with 30 kg/d of milk yield [19], −40°C with 36 kg/d of milk yield [20], or even −45°C [21]. Milk yield could be affected between Holstein and Jersey cows in the range of LCT. Hence, the average milk yield in winter season was increased by 7.2% in Holstein cows compared to the autumn season; with the average THI and Ta being 35.29°C and −1.49°C, respectively. But, no significant difference was observed in Jersey cow’s milk yield between the autumn and winter season.

In particular, the study results showed the different trends in the milk production of Holstein and Jersey cows, when the THI values increased from 16 to 80. A negative regression slope line was observed in Holstein cow’s milk production as THI increased from 16 to 80. Milk yield of Holstein and Jersey cows was reduced slightly (1%) at THI range of 76 to 80 as compared with THI range of 66 to 70. However, milk yields were increased by 15% in Holstein cows at the THI values of 16 to 20, but milk yield was dropped by 11% in Jersey cows. Also, the milk yield dropped by 4% to 5% in Jersey cows, when THI values were between 21 and 50, and a loss of 11% when the THI values reached 16 to 20. Our study results confirmed that the LCT and upper critical temperature affected the milk yield in Holstein and Jersey cows, particularly, Jersey cows experienced more cold stress than Holstein cows during each season in Korea.

The milk fat and protein content increased in Holstein cows when the THI value was 21 to 30 compared to Jersey cows (31 to 35 for milk fat and 26 to 30 for milk protein), and fat and protein content declined in Jersey cows more than in Holstein dairy cows, when the THI value was 76 to 80 and 46 to 50 respectively. Rodriquez et al [22] reported that HS environments have been associated with depressions in milk fat percentage. Bouraoui et al [23] suggested that the depressed fat percentage could be attributed to the decrease in forage intake of the diet, and TMR could alleviate milk fat depression associated with HS by maintaining the intended forage to concentrate intake and, ensuring adequate fiber for proper rumen function. Many studies reported that a decreased milk protein is associated with increased maximum daily temperature [10,13]. The reduction in milk protein is usually caused by a decreased dry matter intake and energy intake [13].

In practice, the yields of milk fat and protein in Holstein and Jersey cows were higher in the winter than in the other seasons. But in Holstein cows, it may be caused by increasing milk yield and milk fat and protein percentages as a result of adaptability to cold stress. However, in Jersey cows, it may be caused by decreasing milk yield as a result of a reduced resistance to cold stress.

## CONCLUSION

According to the results obtained in the study, it was determined there were significant changes in each season’s environment conditions. Also, the results of this study demonstrated that the seasonal changes affected the milk production and composition of Holstein and Jersey cows; also our study established the relationship between the THI and milk production between the Holstein and Jersey cows. Particularly, we observed significantly lowered milk production in autumn season by non-recovered HS from the summer season and high humidity in the autumn season. The milk production was negatively correlated with Holstein cows and positively correlated with Jersey cows with a THI of 16 to 80. No significant changes were seen in milk yield between the Holstein and Jersey cows at THI of 76 to 80. However, milk yield increased by 15% in Holstein cows and declined by 11% in Jersey cows at THI of 16 to 20. In addition, we found significantly higher percentage of milk fat and protein in Jersey cows than in Holstein cows. But, the yield of milk fat and protein content was higher in Holstein cows than in Jersey cows in all the THI ranges. Therefore, this study confirmed that Jersey cows were less adaptable to low temperature of the winter, which resulting in lower milk productivity. It seems to have a negative impact on dairy farmer income under Korea’s milk price estimation system, which places a higher value on milk yield than on milk composition or sanitary grades. Further studies are needed to improve the adaptability of domestic breeding Jersey cows to a low temperature environment.

## Figures and Tables

**Table 1:** Environmental conditions during the experimental periods

Items	Season period			

	Spring	Summer	Autumn	Winter
Ta, minimum (°C)	6.21±0.11^c^	19.57±0.07^a^	9.21±0.17^b^	−7.12±0.11^d^
Ta, maximum (°C)	20.14±0.12^c^	29.31±0.06^a^	20.66±0.16^b^	4.07±0.11^d^
Ta, average (°C)	13.12±0.11^c^	24.02±0.05^a^	14.54±0.16^b^	−1.62±0.09^d^
RH, minimum (%)	29.34±0.31^d^	47.72±0.29^a^	42.87±0.37^b^	35.77±0.34^c^
RH, maximum (%)	87.73±0.59^c^	90.45±0.41^b^	92.11±0.59^a^	85.98±0.75^d^
RH, average (%)	59.75±0.26^d^	71.71±0.21^b^	72.51±0.27^a^	63.89±0.29^c^
Daily THI, minimum	46.72±0.15^c^	65.98±0.10^a^	50.27±0.26^b^	27.21±0.14^d^
Daily THI, maximum	64.06±0.14^c^	76.82±0.08^a^	65.75±0.19^b^	46.01±0.12^d^
Daily THI, average	56.19±0.15^c^	72.55±0.09^a^	58.36±0.24^b^	35.11±0.12^d^

Ta, ambient temperature; RH, relative humidity; THI, temperature-humidity index.

a–d Denotes comparison made within rows (p<0.05).

**Table 2:** The effect of seasonal thermal stress on milk production between the Holstein and Jersey cows

Item	Holstein	Jersey	SEM	p-value
	Milk yield (kg/d per head)			
Spring	30.94^a^,^x^	21.22^a^,^y^	0.17	<0.001
Summer	30.14^b^,^x^	20.96^a^,^y^	0.14	<0.001
Autumn	29.02^c^,^x^	19.75^b^,^y^	0.20	<0.001
Winter	31.12^a^,^x^	19.90^b^,^y^	0.23	<0.001
	FPCM (kg/d per head)			
Spring	29.82^b^,^x^	24.55^a^,^y^	0.20	<0.001
Summer	29.50^b^,^x^	23.34^b^,^y^	0.18	<0.001
Autumn	28.45^c^,^x^	22.96^b^,^y^	0.23	<0.001
Winter	31.08^a^,^x^	22.71^b^,^y^	0.27	<0.001

SEM, standard error of the mean; FPCM, fat- and protein-corrected milk.

a–c denotes comparison made within the same column (comparison between the spring, summer, autumn, winter and Holstein and Jersey cows, respectively) (p<0.05).

x,y denotes comparisons made with same row (comparison with Holstein and Jersey cows in the same season) (p<0.05).

**Table 3:** The effect of temperature-humidity index on milk production and changing rate between the Holstein and Jersey cows based on the temperature-humidity index of 66–70

THI	Holstein		Jersey	
	
	Milk yield (kg)	Change rate (%)	Milk yield (kg)	Change rate (%)
16–20	5.36	15	−2.14	−11
21–25	3.24	10	−0.95	−5
26–30	2.40	7	−1.03	−5
31–35	0.94	3	−0.79	−4
36–40	0.36	1	−1.05	−5
41–45	−0.11	0	−0.71	−4
46–50	−0.22	−1	−0.95	−5
51–55	−0.35	−1	0.02	0
56–60	0.51	2	0.46	2
61–65	0.20	1	0.33	2
66–70	0.00	0	0.00	0
71–75	0.03	0	0.10	0
76–80	−0.42	−1	−0.13	−1

THI, temperature-humidity index; SEM, standard error of the mean.

**Table 4:** The effect of seasonal thermal stress on milk composition between the Holstein and Jersey cows

THI	Holstein	Jersey	SEM	p-value
	Milk fat (%)			
Spring	3.85^b^,^y^	5.36^b^,^x^	0.04	<0.001
Summer	3.93^b^,^y^	4.93^c^,^x^	0.03	<0.001
Autumn	3.91^b^,^y^	5.31^b^,^x^	0.05	<0.001
Winter	4.19^a^,^y^	5.62^a^,^x^	0.04	<0.001
	Milk fat (kg)			
Spring	1.18^b^,^x^	1.10a,y	0.01	<0.001
Summer	1.17^b^,^x^	1.03^b^,^y^	0.01	<0.001
Autumn	1.11^c^,^x^	1.03^b^,^y^	0.01	0.001
Winter	1.28^a^,^x^	1.10^a^,^y^	0.01	<0.001
	Milk protein (%)			
Spring	3.21^c^	3.75^b^	0.02	<0.001
Summer	3.23^c^	3.52^c^	0.01	<0.001
Autumn	3.34^b^	3.88^a^	0.02	<0.001
Winter	3.44^a^	4.00^a^	0.02	<0.001
	Milk protein (kg)			
Spring	0.98^b^,^x^	0.77^a^,^y^	0.01	<0.001
Summer	0.97^bc^,^x^	0.73^b^,^y^	0.01	<0.001
Autumn	0.95^c^,^x^	0.76^ab^,^y^	0.01	<0.001
Winter	1.05^a^,^x^	0.78^a^,^y^	0.01	<0.001
	Solids (%)			
Spring	12.42^c^	14.30^c^	0.05	<0.001
Summer	12.57^c^	13.85^d^	0.04	<0.001
Autumn	12.77^b^	14.60^b^	0.07	<0.001
Winter	13.17^a^	14.96^a^	0.06	<0.001
	Solids (kg)			
Spring	3.81^b^,^x^	2.96^a^,^y^	0.03	<0.001
Summer	3.76^b^,^x^	2.89^ab^,^y^	0.02	<0.001
Autumn	3.65^c^,^x^	2.85^b^,^y^	0.03	<0.001
Winter	4.03^a^,^x^	2.92^ab^,^y^	0.03	<0.001
	Lactose (%)			
Spring	4.73^c^	4.66^b^	0.01	0.001
Summer	4.71^c^	4.72^a^	0.01	0.342
Autumn	4.79^b^	4.69^ab^	0.01	0.007
Winter	4.85^a^	4.68^ab^	0.01	<0.001
	Somatic cell count (×10^3^/mL)			
Spring	111.76^b^	97.19^b^	10.45	0.496
Summer	160.46^a^	140.44^b^	12.11	0.409
Autumn	58.62^c^	275.07^a^	27.83	<0.001
Winter	71.53^c^	61.61^b^	4.06	0.253

THI, temperature-humidity index; SEM, standard error of the mean.

a–d denotes comparison made within the same column (comparison between the spring, summer, autumn, winter season and Holstein and Jersey cows, respectively) (p<0.05).

x,y denotes comparisons made with same row (comparison with Holstein and Jersey cows in the same season) (p<0.05).

**Table 5:** The effect of temperature-humidity index on milk composition between the Holstein and Jersey cows

THI	Holstein	Jersey	SEM	p-value
	Milk fat (kg/d per head)			
21–25	1.30^a^,^x^	1.05abcd,^y^	0.05	0.004
26–30	1.32^a^,^x^	1.12^ab^,^y^	0.03	0.001
31–35	1.28^ab^,^x^	1.15^a^,^y^	0.02	0.011
36–40	1.27^ab^,^x^	1.08abcd,^y^	0.02	<0.001
41–45	1.22^bc^,^x^	1.02bcd,^y^	0.02	<0.001
46–50	1.09^e^	1.03bcd,^y^	0.02	0.106
51–55	1.15cde	1.12abc,^y^	0.02	0.281
56–60	1.18^cd^,^x^	1.08abcd,^y^	0.02	0.002
61–65	1.13^de^,^x^	1.07abcd,^y^	0.01	0.021
66–70	1.16cde,^x^	1.08abcd,^y^	0.01	<0.001
71–75	1.19^cd^,^x^	1.01^cd^,^y^	0.01	<0.001
76–80	1.16cde,^x^	1.00^d^,^y^	0.01	<0.001
	Milk protein (kg/d per head)			
21–25	1.12^a^,^x^	0.77abc,^y^	0.04	<0.001
26–30	1.11^a^,^x^	0.79^a^,^y^	0.02	<0.001
31–35	1.05^b^,^x^	0.79^ab^,^y^	0.02	<0.001
36–40	1.02^bc^,^x^	0.77abc,^y^	0.01	<0.001
41–45	0.98cde,^x^	0.75abc,^y^	0.02	<0.001
46–50	0.93^f^,^x^	0.73^bc^,^y^	0.01	<0.001
51–55	0.97def,^x^	0.78^ab^,^y^	0.01	<0.001
56–60	0.99^cd^,^x^	0.76abc,^y^	0.01	<0.001
61–65	0.97cdef,^x^	0.78^ab^,^y^	0.01	<0.001
66–70	1.00^cd^,^x^	0.76abc,^y^	0.01	<0.001
71–75	0.96def,^x^	0.74abc,^y^	0.01	<0.001
76–80	0.94^ef^,^x^	0.71^c^,^y^	0.01	<0.001

THI, temperature-humidity index; SEM, standard error of the mean.

a–f Denotes comparison made within the same column (comparison between THI and Holstein and Jersey cows, respectively) (p<0.05).

x,y Denotes comparisons made with same row (comparison between the Holstein and Jersey cows in the same THI) (p<0.05).
